# Are all domains created equal? An exploration of stakeholder views on the concept of physical literacy

**DOI:** 10.1186/s12889-022-12931-5

**Published:** 2022-03-15

**Authors:** Sarahjane Belton, Sinead Connolly, Cameron Peers, Hannah Goss, Marie Murphy, Elaine Murtagh, Jennifer Kavanagh, Méabh Corr, Kyle Ferguson, Wesley O’Brien

**Affiliations:** 1 School of Health and Human Performance, Dublin City University, Dublin 9, Ireland; 2 School of Sport, Ulster University, Coleraine, UK; 3 Faculty of Science and Health, Dublin City University, Dublin 9, Ireland; 4 Sport and Exercise Sciences Research Institute, Ulster University, Coleraine, UK; 5 Department of Physical Education & Sport Sciences, Health Research Institute, University of Limerick, Limerick, Ireland; 6 School of Public Health, Physiotherapy, and Sports Science, University College Dublin, Dublin 9, Ireland; 7 Kildare and Wicklow Education and Training Board, Wicklow, Ireland; 8 School of Sport, Ulster University, Coleraine, UK; 9 School of Education, Sports Studies and Physical Education Programme, University College Cork, Cork, Ireland

**Keywords:** Physical literacy, Children, Young people, Consensus statement, Physical education, Physical activity

## Abstract

**Background:**

Developing physical literacy at population levels provides a transformative appeal for those working in sport, health, education, recreation and physical activity settings. Interdisciplinary approaches to development of policy in this area is recommended. The purpose of this study was to gather empirical data from key stakeholders working with young people in areas related to physical literacy across the Republic of Ireland and Northern Ireland, to capture their current understanding and awareness of the physical literacy to help inform the development of the first all-island consensus statement for physical literacy.

**Methods:**

A total of 1,241 participants (52% male), from a range of stakeholder groups (health, physical activity, sport, recreation and education) completed a researcher developed physical literacy questionnaire. A one-way MANOVA was carried out to investigate differences across stakeholder grouping in terms of perceived importance of three domains of physical literacy. Overlap of independent confidence intervals was analysed to determine importance of the physical literacy domains within stakeholder grouping.

**Results:**

A majority (63%) of respondents indicated they were aware of an existing definition of physical literacy, but this varied by stakeholder group (e.g. 86% for higher education, versus 47% of coaches). Participants working in higher education (69%), or working as physical education specialists (67%), were more likely to rate themselves as experts or near experts in physical literacy, while coaches, education generalists, and decision makers were more likely rate themselves as having no expertise (9%, 12% and 12% respectively). Non-specialist teachers and physical education teachers rated the importance of all domains of physical literacy significantly higher than decision makers, and significantly higher than coaches in the cognitive and affective domains. All stakeholders significantly rated the importance of the physical/psychomotor domain of physical literacy higher than the affective or cognitive domains of physical literacy.

**Conclusions:**

Differences observed across stakeholder groups underline the importance of developing a shared vision for physical literacy, and the need to clarify and gain consensus on a definition of the term and its domains. Engaging and understanding the voice of stakeholders is critical in ensuring the relevance, ownership of and commitment to physical literacy statement operationalisation.

## Background

Physical literacy is a concept which has gained increased attention in recent years, with many countries working to develop and release consensus statements regarding the definition of the concept, including Australia and Canada. Margaret Whitehead’s work [1, 2] provides the basis for the development of many of the emerging definitions. Consistently, literature relating to physical literacy refers to the lifelong journey individuals undertake to engage in and maintain physically active lifestyles [3]. A stated goal for developing physical literacy is to help an individual develop and maintain the appropriate skills, knowledge and attitude required to live a healthy, active lifestyle [3]. An examination of international physical literacy statements, frameworks and policy documents shows that physical literacy is cited as being important for the achievement of lifelong physical activity and sport engagement [4–7]. Low levels of physical activity in young people and adults are a concern in both the Republic of Ireland (RoI) and Northern Ireland (NI). Recent data from the 2018 Children’s Sport Participation and Physical Activity (CSPPA) study show that only 13% of children aged 10 to 18 years in RoI and NI meet the physical activity guidelines of at least 60 minutes/day of moderate-to-vigorous PA every week [8].

A recent systematic review by Edwards et al. noted that physical literacy was being heavily promoted in sport, health-related physical activity and recreation contexts, but each offered various representations of the concept and limited consensus on its central tenets (definition, philosophical assumption and expected outcomes) [9]. Critics have also argued that physical literacy is not a new concept, and is merely another fad term, with many components of physical literacy already developed in existing related fields, for example, through high quality physical education (PE) [10–12]. Advocates for physical literacy, however, place the concept as a holistic, overarching, umbrella term for a range of components, that Dudley et al [13] posit can be “encouraged, acquired, developed and sustained” (pp 449) across a range of social contexts and sectors; not just within an educational context [14]. The current understanding of these components, and the overall concept, however, has limited empirical evidence and is fraught with confusion. The debate in the literature about ‘what physical literacy is’ means that to date, the research base, the attempts to assess, and the potential impact of the operationalisation of physical literacy, have all been hampered, critiqued and debated [11, 15, 16].

In order to confirm the claims made about the potential for physical literacy, and to better understand its determinants and correlates, and how physical literacy can be effectively operationalised, clarity and agreement on its definition is needed [15, 17, 18]. Researchers have called for transparency in this process [12, 14], as well as greater accessibility for practitioners in terms of language [17, 19]. Such clarity is of crucial importance when developing consensus or position statements. In the RoI and NI to date, without an agreed position or consensus around the concept, physical literacy has been developed sporadically across a range of sectors, without the benefit of cross-sectoral strategic direction. Many people working across related fields may already promote components of physical literacy throughout their work, but to date these stakeholders have had little to no input in the evolution of physical literacy. Much of the existing physical literacy policy internationally has adopted similar top-down approaches, which some authors have suggested has limited the impact of the concept in practice [20]. Dudley et al [13] advocate for ‘genuine engagement in the co-production of policy’ and the devolution of decision making and power to panels of stakeholders. Even more recently, a critical reflecting on the implementation of physical literacy policy in New Zealand/ Aotearoa, Stevens et al. [21] invites researchers to be more considerate of the cultural and social context in which policy is implemented. Arguably, incorporation of the voices of stakeholders in the development of policy position (bottom-up) offers an opportunity to produce a more nuanced strategic position, and a policy that is more relevant to the social and cultural context in which it will be implemented. In turn this can help ensure that those on the ground, who will drive its operationalisation, will be more engaged and committed to its success.

The development of an all-island statement on physical literacy represents a logical first step towards the development of physical literacy policy that will lead to physical literacy being theoretically understood, and practically employed in a strategic and coherent way, across a range of sectors and populations on the island of Ireland. As part of a broader piece of research commissioned by Sport Ireland and Sport Norther Ireland, the purpose of this study was to gather empirical data from key stakeholders from across the island of Ireland (those working with young people) on their current understanding, and awareness of the construct of physical literacy to inform the development of the first all-island consensus statement for physical literacy, and to offer guidance for the subsequent strategic /policy direction and implementation.

## Methods

### Participants and recruitment

The target cohort of this study were stakeholders from across the island of Ireland working or volunteering with young people in areas related to physical literacy; including sport, education, physical activity, recreation, health. The decision to focus the research on this cohort was taken, in consultation with Sport Ireland, as it was considered that this cohort were likely to be those most involved in operationalisation of the all-island consensus statement across the island of Ireland. Participants were recruited to this study through the networks of contacts that the research team had in their individual institutions, along with the network of contacts existing through Sport Ireland (SI) and Sport Northern Ireland (SNI) databases and networks. The questionnaire, which included a plain language statement and consent form, was disseminated electronically directly by members of the research team, SI and SNI, to their networks. Recipients were also asked to further share it with others across RoI and NI whom they knew worked or volunteered in an area related to PL. Ethical approval for this study was granted by the University of Ulster School of Sport Filter Committee (FC06 2019-20).

### Questionnaire

A researcher developed questionnaire was created through consultation with all members of the research team along with SI and SNI, to ensure that questions captured the key areas required to help inform development of Ireland’s all island statement on physical literacy. The questionnaire was piloted with a small group prior to national dissemination, to ensure readability and appropriate timing, with some minor changes subsequently made to wording. The pilot group consisted of a convenience sample of 20 people working or volunteering with young people in areas related to physical literacy. The questionnaire was completed online by participants and took approximately 10 minutes to complete. Questions relating to participant demographics, including gender, age, and the role/area in which participants work (as it related to physical literacy) were included at the start of the questionnaire. Respondents were then invited to respond to an open-ended (optional) question; ‘Definitions of physical literacy vary across the world depending on the specific environment and context. What does Physical Literacy mean to you?’. The purpose of this question was to allow participants the opportunity to articulate potential components of physical literacy that may be important to them, but that may not have been included later in the closed questions later in questionnaire (where a list of potential components was given, see Table 1). Critically, this question was asked prior to sharing a list of potential components of physical literacy with participants (see the third question shown in the list below). The qualitative responses to the open ended question were thematically analysed to identify whether any potential additional components of physical literacy (additional to those shown in Table 1) were present in the responses.

**Table 1:** Components of Physical Literacy included in the Questionnaire, and Categorisation of same into Domains

Physical/Psychomotor	Cognitive	Affective
Physical Activity	Understanding how to move in Physical Activity and Sport	Motivation
Motor Competence	Knowledge of Physical Activity	Self-Efficacy
Fundamental Movement Skills	Knowledge of Movement	Confidence
Physical Fitness	Knowledge of Awareness of Importance of Physical Activity for Health	Self-Competence
Positive Physical Activity Behaviours	Understanding how to improve in Physical Activity and Sport	Valuing Physical Activity
Engaging in Movement	Creativity in a range of Physical Activity and Sport	Enjoyment
	Responsibility for own participation in Physical Activity and Sport	Interaction with others in Physical Activity in Sport
		Resilience
		Physical Activity Attitudes

Participants were then asked the following closed questions relating specifically to physical literacy including;

- Are you aware of any existing definitions of physical literacy? (Yes of No)
- How would you rate your expertise in the area of physical literacy? (0=None at all, 5= I consider myself an expert)
- Consider the following list [see Table 1 for the list shown] and rate your views on the importance of each as a component/element of Physical Literacy (1 ‘*Not a component of Physical Literacy’*, 2 ‘*Small Component of Physical Literacy’*, 3 ‘*Important Component of Physical Literacy’*, and 4 ‘*Vital Component of Physical Literacy’*).

The components of physical literacy included in the questionnaire, as shown in Table 1, were identified, through a rapid review carried out by the research team, as those that had been associated with different definitions of physical literacy across the literature internationally. The open ended question posed prior to this ‘What does physical literacy mean to you?’ ensured that the research team could also identify any additional components that may be culturally important to this RoI and NI cohort, but had not come up in the international literature.

### Data processing

Participants work role was collapsed into five broad categories as shown in Table 2; Higher Education (Researcher and College/University Lecturer), Education (Preschool Teacher, Primary School Teacher, Post Primary (non-PE) Teacher, and School Principal), Coach, PE Teacher, and Decision Maker (Programme Manager/Lead, Sports Leader, Sports/Physical Activity Coordinator, and Service Provider). The scores for each individual’s ranking of the 22 physical literacy components were summed, and a mean (SD) score for each component was calculated (Table 3). Additionally, in line with Whitehead’s conceptualisation [10, 17, 22], and as applied across a range of physical literacy research internationally [3, 4, 18, 20, 21], the 22 components were also categorised into the three broader domains of Physical/Psychomotor, Cognitive, and Affective [10, 17, 22] , as shown in Table 1. The scores for each participant’s perceived importance of the individual components were summed and averaged, with a mean score for each domain calculated for each participant (Table 3).

**Table 2:** Awareness and Expertise of Physical Literacy held by respondents (across role type)

	Awareness (*n* = 909)	Perceived Expertise (*n* = 844)	Role title of respondents	n	% of Total
**Coach**	**47%**	39%	Coach/Instructor	345	33.3
**PE Teacher**	**74%**	67%	Post Primary PE teacher (specialist)	205	19.8
**Decision Maker**	70%	40%	Programme Manager/Lead	125	12.1
			Sports/Physical Activity Coordinator	87	8.4
			Service Provider	38	3.7
			Sports Leader	41	3.9
			Advocate	12	1.2
**Higher Education**	**86%**	69%	College/University Lecturer	64	6.2
			Researcher	15	1.5
**Education**	**53%**	38%	Primary Teacher	62	6
			School Principal	32	3.1
			Post Primary (non PE) teacher	7	0.7
			Pre-school Teacher	1	0.1

Awareness = Aware of any existing definitions of physical literacy

Perceived Expertise = indicating their expertise as 4 or 5 (out of 5) in physical literacy

**Table 3:** Perceived Importance of Potential Components of Physical Literacy

Physical Literacy Components	Mean	SD
Physical Activity	3.644	0.606
FMS	3.620	0.617
Enjoyment	3.525	0.718
Motor Competence	3.376	0.706
Understanding how to move	3.355	0.668
Engaging in Movement	3.347	0.656
Valuing PA	3.285	0.784
Motivation	3.256	0.784
Knowledge and Awareness of Importance of PA for Health	3.206	0.772
Positive PA Behaviour	3.203	0.725
Confidence	3.176	0.781
Responsibility for participation in PA and Sport	3.172	0.779
Self Efficacy	3.066	0.771
PA Attitudes	3.045	0.773
Self Competence	3.042	0.761
Interaction with others in sport	3.002	0.818
Understanding how to improve PA and Sport	3.000	0.759
Resilience	2.895	0.814
Creativity range of PA and Sport	2.891	0.800
Knowledge of Movement	2.865	0.817
Knowledge of PA	2.834	0.822
Physical Fitness	2.713	0.816
**Physical Literacy Domains**		
Physical/Psychomotor	3.3171	0.45444
Affective	3.1435	0.55819
Cognitive	3.046	0.55149

### Data analysis

#### Between groups

Descriptive statistics (mean, SD, confidence intervals, and cross tabulations) were calculated for all physical literacy components and domains. To understand if there was an interaction between stakeholder groups a one-way multivariate analysis of variance (MANOVA) was run with stakeholder group as the independent variable and physical literacy domains (physical/psychomotor, cognitive, and affective) considered separately as three dependent variables. Follow up analyses were conducted using univariate two-way ANOVAs and the main effect of gender and stakeholder were considered separately. Tukey pairwise comparisons were run for stakeholder and gender, when necessary, to highlight differences in physical literacy domain importance. A one-way MANOVA was chosen over a series of ANOVAs for protection against inflated Type I error due to multiple testing [23]. Bonferroni adjustments were further carried out to further protect against Type I error. Effect-size measures were presented for the comparison analyses, considering partial η2 ≥ 0.01, partial η2 ≥ 0.06 and partial η2 ≥ 0.14 as small, medium and large effects, respectively [24].

#### Within groups

For a statistically significant comparison of domains, (e.g., importance of physical/psychomotor vs importance of affective), upper and lower confidence intervals were compared; those that overlapped by less than 50% (*p* < .05) were considered statistically significant, and referred to as a proportional overlap [25, 26]. In addition, when the two CIs did not overlap, the proportional overlap was considered a proportional gap (*p* < 0.001). Simply, if the upper confidence interval of the smaller mean does not extend 50% over the lower confidence interval of the greater mean, then statistical difference at the *p* < .05 level is observed [25, 26].

#### Qualitative

Responses to the open-ended question were analysed by the lead and fourth author, both experienced mixed methods researchers, using reflexive thematic analysis [27, 28]. It is inevitable that potential biases exist. Both colleagues are heavily involved in physical literacy related work, having worked for many years with young people as both researchers and practitioners in the area of physical literacy across a range of domains including teaching, research and sport coaching. Across this work, both researchers have consistently promoted and considered all domains of physical literacy equally in terms of their importance. Both identify themselves as pragmatic researchers. To limit the impact of any potential biases, specific steps were taken in qualitative analysis of this open-ended question. The purpose of the question was to gain insight into any additional components of physical literacy not already included within existing conceptualisations (see Table 1), and as a result analysis was guided by existing knowledge. The lead author independently coded answers and generated initial themes. These themes were then presented to the fourth author who acted as a ‘critical friend’ to prompt reflection in the lead author, and to collaborate to review, define, and name themes [28, 29]. Trustworthiness of the data analysis was further developed using triangulation with the quantitative analysis conducted in this study (which was necessary given the purpose of the open ended question), and ongoing critical reflection of researcher engagement with the analysis process; providing the opportunity to explore, challenge, and extend interpretations [28, 29]. The lead authors also presented themes and verbatim response text to co-authors, as a further means of triangulation [30].

## Results

### Participant overview

A total of 1,241 participants completed the questionnaire between December 2019 and January 2020. Participants ranged in age bracket from 13 – 18 years (3%), to 55+ years (16%); 6% 19 – 24 years, 11% 25 - 30 years, 15% 31-36 years, 20% 37 – 42 years, 14% 43-48 years, and 15% 49 – 54 years. Participants reported as 52% male, 47.7% female, and 0.3% non-binary gender. Most respondents reported working in the RoI (54%), with 24% working in NI, and 22% working across both jurisdictions. The greatest proportion of respondents indicated they worked in urban areas (30%), followed by rural areas (23%) and then suburban areas (19%), with 28% of the respondents indicating they work across all three areas.

### Physical literacy roles held by respondents

As shown in Table 2 below, participants (*n* = 1034 for this question) identified as holding a range of different roles relating to PL with over 50% of the respondents identifying themselves as post-primary PE teachers, or coaches/instructors. A large percentage of participants indicated they work with post-primary school aged children (84%), with 63% indicating they work with primary school aged children, and 19% working with preschool aged children (again noting participants could select more than one response option as appropriate).

### Awareness of existing definition, and perceived expertise in physical literacy

When considering awareness of an existing definition of physical literacy (respondents to this question *n* = 909), 63% of respondents indicated that they were aware of a definition (see Table 2). Participants’ perceived expertise varied, with 10% (of respondents to this question, *n* = 844) identifying as ‘experts’ in physical literacy, and 9% indicating they have no expertise. This perceived expertise level varied across work grouping with 1% of those in Higher Education and 3% of those in PE rating their expertise as ‘Not at all’, while these figures were higher for the other domains (9% for Coaches, and 12% for both Education and Decision Makers).

### Ranking of importance of physical literacy components

**Fig. 1:**
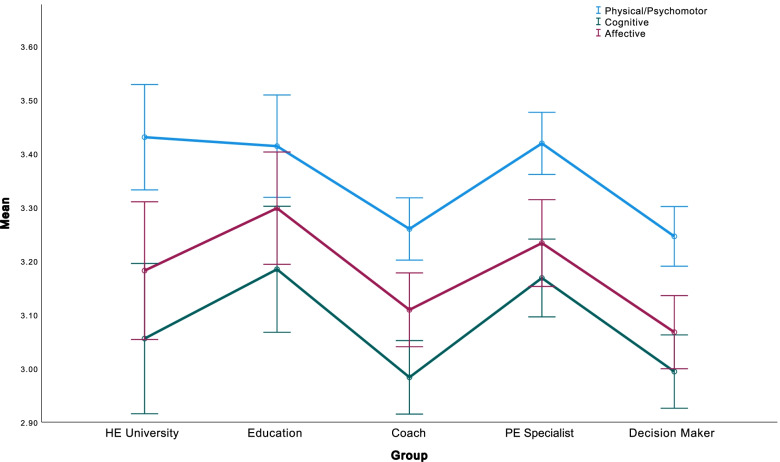
Mean (95% confidence intervals) perceived importance of physical literacy domains between stakeholder groups.

Participants ranking of the perceived importance of the potential components of physical literacy is shown in Table 3 below (*n* = 913). Figure 1 displays the breakdown of mean scores (with 95% confidence intervals) across the composite variables of Physical/Psychomotor, Affective and Cognitive domains, across the five stakeholder groups (Higher Education, Education, Coach, PE Teacher, and Decision Maker).

### Between stakeholder groups: differences in perceived importance

#### One-way MANOVA: assumptions

There was a linear relationship between the dependent variables, as assessed by scatterplot. There was no evidence of multicollinearity, as assessed by Pearson correlation (|*r*| < 0.7). There were 42 univariate outliers in the data, as assessed by inspection of a boxplot for values greater than 1.5 box-lengths (34 univariate outliers) and 3 box-lengths (8 extreme univariate outliers) from the edge of the box. There were ten multivariate outliers in the data, as assessed by Mahalanobis distance compared against a chi-square (χ2) distribution 16.27 (*p* > .001). All outliers were assessed for data entry and measurement errors and deemed genuinely unusual values [31]. The three physical literacy domains were not normally distributed, as assessed by a Bonferroni corrected Shapiro-Wilk's test (*p* < .05). The one-way MANOVA is robust to deviations from normality with respect to Type I error [32]. Moreover, Weinfurt [33] notes that in practice MANOVAs should be performed even if the data is not normal due to a consensus that MANOVA is robust to non-normality. There was homogeneity of covariance matrices, as assessed by Box's M test (*p* = .029), and homogeneity of variances, as assessed by Levene's Test of Homogeneity of Variance (*p* > .05).

#### One way-MANOVA: results

There was a statistically significant stakeholder group (*N* = 5) effect on the combined dependent variables (physical literacy domains), F(12, 2535) = 2.924, *p* < .001, Pillai’s Trace = .041, partial η2 = .014. There was a statistically significant main effect of stakeholder for the three physical literacy domains; Physical/Psychomotor (F(4, 845) = 6.741, *p* < .001, partial η2 = .031), Cognitive (F(4, 845) = 4.507, *p* = .001, partial η2 = .021), or Affective (F(4, 845) = 4.582, *p* = .001, partial η2 = .021). Table 4 below displays the Univariate Effects for Physical Literacy Stakeholders; data are expressed as mean with 95% confidence interval. Tukey post-hoc tests showed that for perceived importance of the physical/psychomotor component, HE University, Non-specialist teachers, and PE Teachers had statistically significantly higher mean scores than Coaches (*p* < .0005) or Decision Maker (*p* < .0005). For Cognitive scores, Tukey post-hoc tests (see Table 5) showed that Non-Specialist Teachers and PE Teachers had statistically significantly higher mean scores than Coaches (*p* < .0005) or Decision Maker (*p* < .0005). For Affective scores, Tukey post-hoc tests showed that Non-Specialist Teachers and PE Teachers had statistically significantly higher mean scores than Decision Maker (*p* < .0005), and Non-Specialist Teachers had statistically significantly higher mean scores than Coaches (*p* < .0005). Table 5 displays the comparisons of stakeholder importance for the three separated physical literacy domains, the difference between stakeholder means, and significance for each comparison (*indicates the mean difference is significant at the 0.01 level).

**Table 4:** Significant univariate effects for Physical LiteracyStakeholders

								Bootstrap 95% Confidence Interval (10,000 samples)	
Dependent Variable	Df	Df Error	F	ηp2	Stakeholder	Means	SE	Lower Bound	Upper Bound
Physical/ Psychomotor*	4	845	6.741	0.031	HE University	3.432	0.05	3.329	3.534
					Non-Specialist Teacher	3.43	0.05	3.337	3.523
					PE Teacher	3.416	0.03	3.35	3.481
					Coach	3.277	0.03	3.222	3.331
					Decision Maker	3.247	0.03	3.191	3.303
Cognitive*	4	845	4.507	0.021	HE University	3.056	0.07	2.941	3.191
					Non-Specialist Teacher	3.196	0.06	3.083	3.309
					PE Teacher	3.163	0.04	3.083	3.242
					Coach	3.003	0.03	2.937	3.069
					Decision Maker	2.997	0.03	2.929	3.066
Affective*	4	845	4.582	0.021	HE University	3.189	0.05	3.063	3.316
					Non-Specialist Teacher	3.314	0.05	3.199	3.416
					PE Teacher	3.227	0.04	3.146	3.307
					Coach	3.112	0.04	3.045	3.179
					Decision Maker	3.069	0.04	3	3.138

Breakdown of follow-up univariate ANOVAs; * *p* <. 001

**Table 5:** Mean difference of each stakeholder groups Perceived Importance across separate Physical Literacy Domains

		HE University	Non-Specialist Teacher	Coach	PE Teacher
Non-Specialist Teacher	Physical/Psychomotor	0.0006			
	Cognitive	-0.1425			
	Affective	-0.131			
Coach	Physical/Psychomotor	.1709*	.1703*		
	Cognitive	0.0721	.2146*		
	Affective	0.073	.2040*		
PE Teacher	Physical/Psychomotor	0.0111	0.0104	-.1598*	
	Cognitive	-0.1162	0.0263	-.1883*	
	Affective	-0.0539	0.0772	-0.1269	
Decision Maker	Physical/Psychomotor	.1844*	.1844*	0.0142	.1740*
	Cognitive	0.0621	.2046*	-0.01	.1783*
	Affective	0.1162	.2472*	0.0432	1700*

Posthoc comparison of stakeholder value using Tukey-Kramer. Mean difference shown in each case, *shows the mean difference is significant at the 0.01 level

### Within stakeholder groups: Differences in perceived importance

**Fig. 2:**
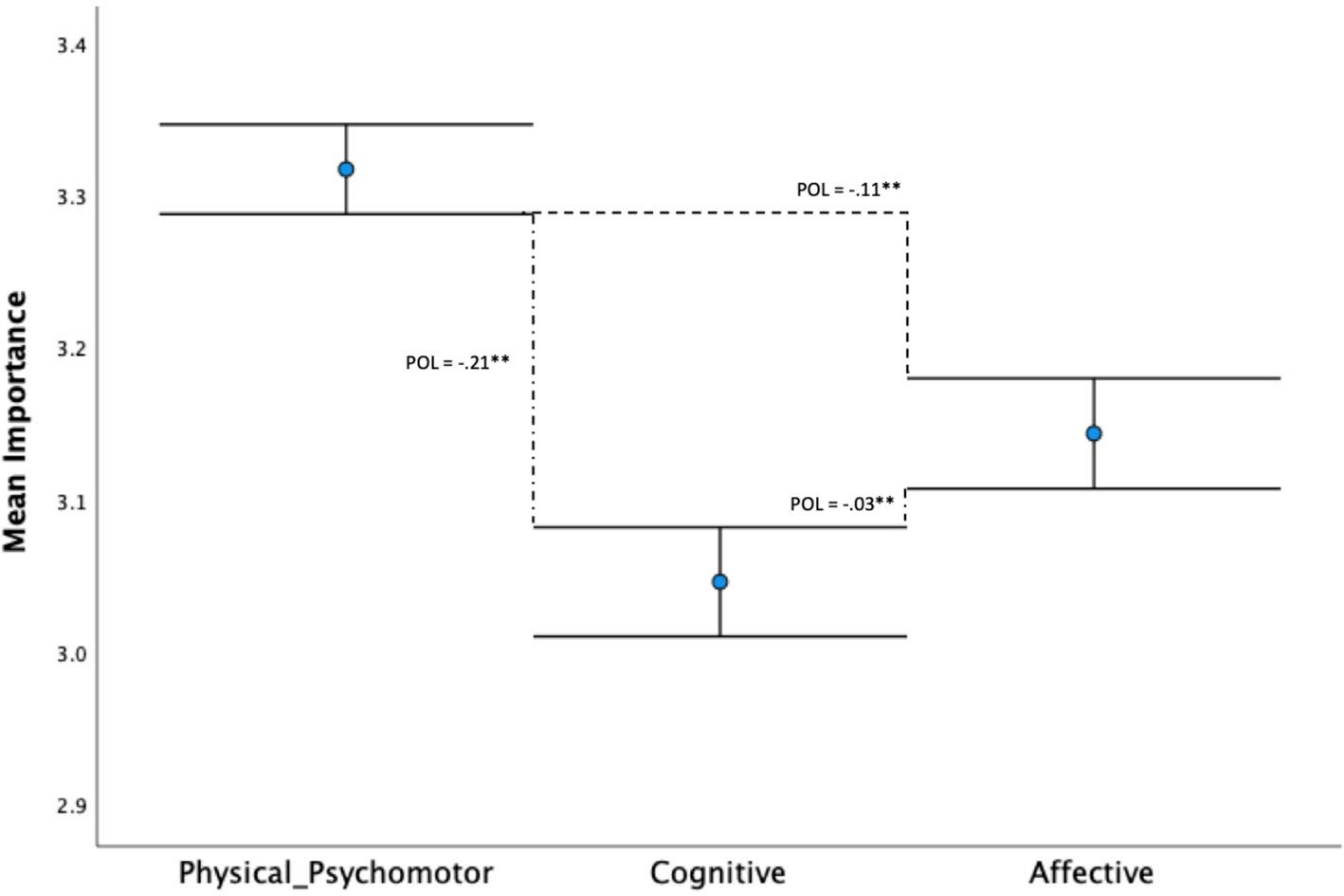
Mean comparison of (95% confidence intervals) perceived importance of physical literacy domains. Note. Proportion overlap (POL) is the overlap expressed as a proportion of the length of a single arm of a CI. POL values are shown alongside difference line. A gap between intervals is signalled by a negative POL value. ** p <. 001 and * p <. 01

**Fig. 3:**
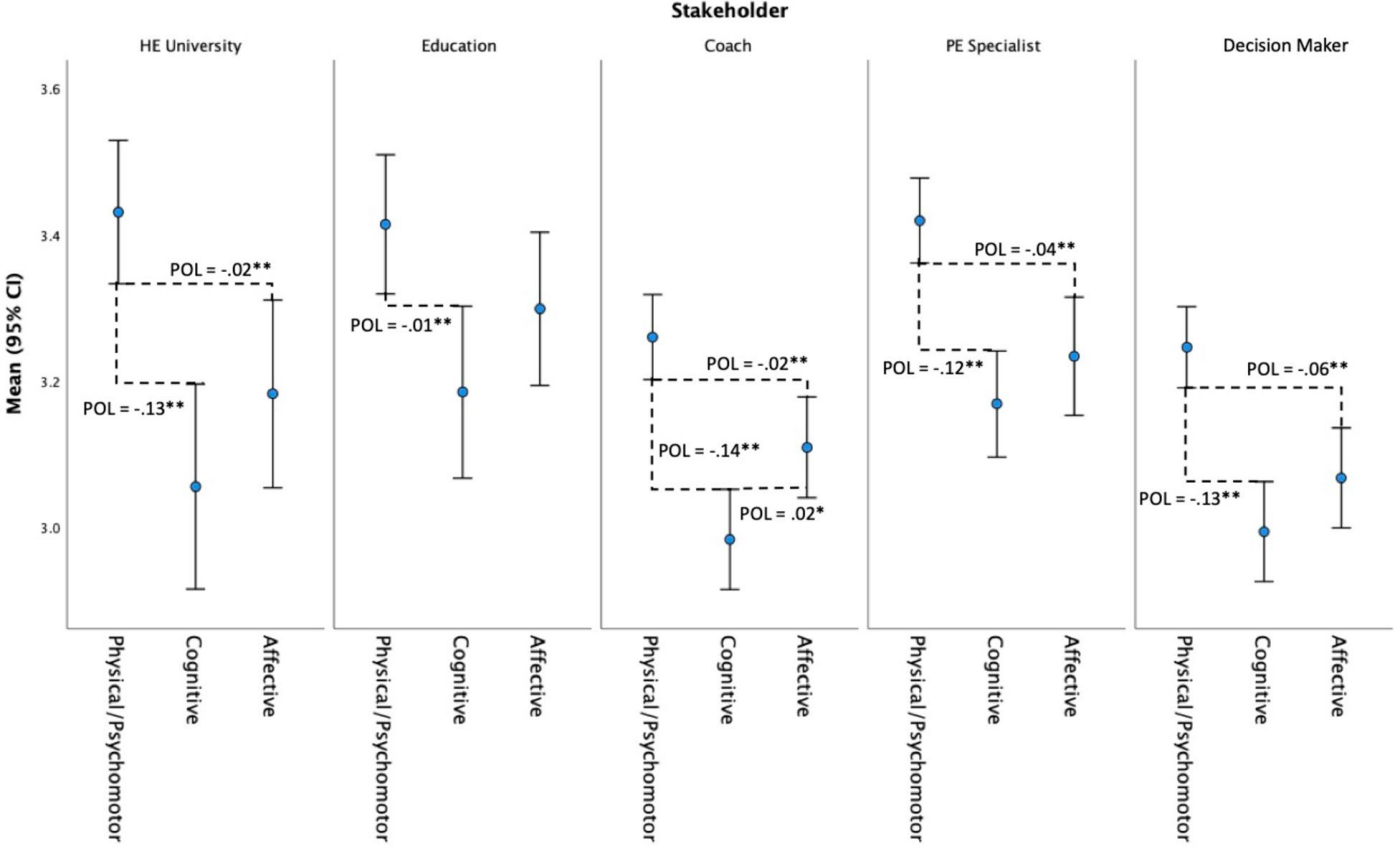
Mean comparison (95% confidence intervals) of perceived importance of physical literacy domains within stakeholder groups. Note. Proportion overlap (POL) is the overlap expressed as a proportion of the length of a single arm of a CI. POL values are shown alongside the difference line. Proportion gap between intervals is signalled by a negative POL value. ** *p* <. 001 and * *p* <. 01

The mean of the three physical literacy domains (Physical/Psychomotor, Cognitive, and Affective) were compared for population, and subsequently for stakeholder. The corresponding 95% confidence intervals were estimated via bias corrected bootstrap (10,000 re-samples). A proportion gap (*p* < .001) was observed between all three domains of physical literacy at the population level (see Fig. 2). At the stakeholder level, results show a proportion gap between the Physical/Psychomotor domain lower confidence interval and the Affective and Cognitive domain upper confidence intervals for all stakeholders (see Fig. 3). This indicates that all stakeholders significantly rated the importance of the Physical/Psychomotor domain of physical literacy higher (*p* < .001*)* than the Affective or Cognitive domains of physical literacy. In addition, the only stakeholder group that placed significantly higher importance on the Affective domain of physical literacy the Cognitive domain was Coaches, as highlighted by a proportion overlap of the affective lower confidence interval and the cognitive upper confidence interval (POL = .02, *p* < .01).

### Qualitative: What does physical literacy mean to you?

A total of 597 participants responded to the final open-ended question, inviting expressions of what physical literacy meant to them. Nineteen components were identified from the data, which were judged by the researchers to be sufficiently distinct from the components of physical literacy that had been indicated in the questionnaire as shown in Table 1. These were captured under four broad themes; social benefits, movement vocabulary and safety, lifelong journey, and personal benefits. Details of the components under these four themes are shown in Table 6 below.

**Table 6:** Themes highlighting potential additional components of physical literacy

Social benefits	Movement vocabulary and safety	Lifelong journey	Personal benefits
Social skills	Language of sport	Life span	Mindful and aware of the body and confident
Relate well to others	Self-awareness of one’s own body	Respond to the demands of life	Mental attitude and strength
Competent within society for life	Ability to move your body effectively in order to carry out tasks and avoid injury	Philosophy of movement and activity	Body Mind awareness
Participate in a meaningful way in society	Body's needs and limits to enjoy physical activity safely	Better life choices	Emotional and cognitive benefits
Become a better citizen	Achieve optimal movement		Personal development and fulfilment

These themes were i) Social benefits, representing a variety of interpersonal and other skills,(e.g. *‘To become a better citizen*’; ‘*They will be able to relate well to others’* ii) Movement vocabulary and safety, with particular consideration of safe and effective movement (e.g. ‘*Having an understanding of the body's needs and limits to enjoy physical activity safely*’; ‘*Ability of the human body to achieve optimum movement considering participants age and ability’*. iii) Lifelong journey, representing the value and impact of physical literacy across the lifecourse, (e.g. ‘*A lifelong journey of being physically active in terms of active living, recreational activity and organised sport; ‘enables one to value and participate in a meaningful way in a society over a lifetime*’, and iv) Personal benefits, representing an holistic appreciation of the innate value of physical literacy for an individual (e.g. ‘*To be mindful and aware of the body and confident because of this*’; ‘*the need and aspiration to reach one physical capability through exercise, movement, training and is necessary for true personal development/fulfillment*’).

## Discussion

This study set out to gather empirical data from key stakeholders working with young people in areas related to physical literacy across the Republic of Ireland and Northern Ireland, to capture their current understanding and awareness of physical literacy in order to inform the development of the first all-island consensus statement for physical literacy, and to guide strategic /policy direction and implementation from the bottom up. Acknowledgement and recognition of specific cultural contexts and social inequalities are identified by Dudley et al. [13] as being a central pillar to effective policy development in physical literacy. As a result, differing international approaches to physical literacy have emerged in the interpretation and operationalisation of physical literacy, although this itself is not without debate. Most recently, a paper by Stevens et al. [21] presented the concerns of the impact of a Westernised interpretation of physical literacy that neglects the values, identity and aspirations of Aotearoa New Zealand.

While physical literacy has the potential to be a unifying concept that can enable stakeholders from health, sport and education to strategically come together [5, 6, 13, 34], in Ireland this practice is only emerging. Dudley et al. [13], in a paper on critical considerations for physical literacy policy development, suggested that those working to embed physical literacy within policy should engage the various agencies and co-create the major shift needed to embed physical literacy across sport, recreation, education, health, planning and transport. The authors further highlight that the development of physical literacy is not individualistic, but rather the result of social processes, and is evolved through practice embedded in social and cultural contexts, underlining that those working within and across the sectors of education, recreation, sport and health are important agents [13]. Crucially, the current paper is one of the first to include and synthesise the stakeholder voice from across these sectors in relation to physical literacy. As such it acknowledges the importance of this cross-sectoral approach to help build an understanding of the Irish landscape as a foundation for the development of the physical literacy statement.

### Physical literacy awareness and expertise

Interestingly, while the majority of respondents (89%) indicated they work in an area directly related to physical literacy, just 43% considered themselves experts or near experts in the area of physical literacy. Unsurprisingly, and consistent with the findings by Goss et al.in a sample of ‘academic/practitioner experts’ [35], those working in higher education (69%) or as PE specialists (67%) were more likely to rate themselves as experts or near experts, while Coaches, Decision Makers and those working in education more generally were more likely to indicate they had no expertise (9%, 12% and 12% respectively). This presents a potential challenge to policy development and implementation where those in a position to advocate for PL, due to their role as decision-makers, or their role as providers of PL programmes to young people in education and sport, feel least expert in this field. This also underlines that current explanations and communications around physical literacy may not be sufficient for the range of contexts in which physical literacy is acquired, developed and promoted, and efforts to ‘demystify’ physical literacy, and build confidence and expertise in specific groups will be needed. As a result, future work needs to be better translate theory around physical literacy in a way that is accessible to a range of audiences; which is especially pertinent given that one of the most unique aspects of physical literacy is its application across a number of disciplines. The link between physical literacy theory, understanding, policy and practice is reciprocal, and consideration of all of these levels is needed to advance the field. Consistent with Dudley et al’s [13] contention that agency executives have a major leadership responsibility to play in devolving power and decision making to panels of stakeholders with whom they engage, Irish ‘decision makers’ working to embed physical literacy within policy, and to operationalise the consensus statement on physical literacy, may benefit from engaging fully with others who have expertise in the area for support and insight. Communication between decision makers and PE teachers may be particularly critical, with research suggesting that teachers are often left out of significant and strategic decision making, and not consulted or provided with the opportunity to give input or feedback [36].

### Perceived importance of physical literacy components and domains

Mindful of the concerns raised in previous research regarding the over-simplification of physical literacy [11, 17], or that components hold different meanings to different groups [5, 17, 18], this study sought to explore a broad range of components, and also an opportunity for participants to articulate what physical literacy means to them in their own words. The three domains selected to organise the physical literacy components in this paper (Physical/Psychomotor, Cognitive and Affective), are those consistently used throughout physical literacy research [3, 5, 14, 37–41]. Though there is variation in the perception of importance or ‘value’ of physical literacy domains across stakeholder groups, it is true to say that the data presented in Table 3 suggests that all the components listed are considered in some way ‘important’ by participants in this study to a greater or lesser extent. Physical Fitness, Knowledge of Physical Activity, Knowledge of Movement, Creativity in a Range of Physical Activities and Sports and Resilience were the lowest ranked, with means ranging from 2.7 to 2.9 (with 3 being the ranking for an ‘*Important Component of Physical Literacy’)*. All other physical literacy components scored a mean of 3 or above, with Physical Activity, Fundamental Movement Skills and Enjoyment ranking highest.

Different stakeholder-perspectives are apparent in the results, with those working in an educational paradigm (non-specialist teachers and PE Teacher alike) scoring all three domains of physical literacy significantly higher in importance than decision makers, and significantly higher than coaches in the Cognitive and Affective domains. The higher level of importance placed on physical literacy domains by this cohort may be attributed to initial teacher education, which is known to play a role in shaping teachers beliefs [42], and encourage holistic approaches to child development [43, 44]. While all stakeholders will need to work collaboratively operationalise physical literacy, it is the coaches and those in decision making roles that may need more convincing about its importance.

### Priority of physical literacy domains within stakeholders

For the most part the Affective and Cognitive domains are held in equal importance *within* each Stakeholder group, with the exception being the Coaches group, who place significantly higher importance on the Affective domain than the Cognitive domain. Results of the within-stakeholder analysis in this study indicate however that all stakeholder groups rated the importance of the Physical/Psychomotor domain of physical literacy significantly higher than the Affective or Cognitive domains. Work carried out in Canada, which used factor analysis to help refine the Canadian Assessment of Physical Literacy (CAPL), found the cognitive domain was deemed to have lower relative importance to physical literacy assessment overall, which led to the revision of the Knowledge and Understanding (Cognitive) domain within CAPL-2 [45]. CAPL-2 and the present study both reflect a youth context, as such it could be considered that in this life stage a difference in the relative importance being placed by stakeholders on the physical/psychomotor domain over other domains may exist, which may have implications for the operationalisation of physical literacy in this age group specifically. Findings of this study are consistent with some of the academic literature to date which has prioritised the physical/psychomotor domain (for recent examples, see Said [46] and Warner [47]), albeit against Whitehead’s conceptualisation that all domains are equally important [37].

It could be argued that the perceptions of participants in the current study simply reflect a lack of understanding of the holistic nature of the concept, which has been observed in previous research in early years [48], school [35], and health settings [49]. While the perceptions of participants should not be diminished or discounted, the prioritisation of the physical domain within physical literacy has been an ongoing area of contention in the field (e.g .[50]), with some suggesting that the prioritisation of this domain leads to a narrow, dualistic, understanding that suggests becoming ‘physically literate’ as an end state outcome [9, 15, 22, 51], and moves away from monism; a philosophical underpinning of physical literacy [1, 52]. This evidence of current understanding, presents the need for further education opportunities to convey a deeper, holistic conceptualisation of physical literacy across, within, and between contexts and stakeholders. In practical terms, it points to the potential gains to be made in developing targeted education strategies when working with stakeholder groups in the context of youth physical literacy. For example, in recognising the dominance of the physical domain within each stakeholder group, it is recommended that future education campaigns with such stakeholders would emphasise the breadth of domains of physical literacy, reinforce the holistic nature of the concept, and underline the importance of developing across all domains.

### ‘What does Physical Literacy mean to you?’

As well as ranking the importance of the dimensions included in physical literacy, participants were asked, in an open-ended question (‘What does Physical Literacy mean to you?’), about their own interpretation of physical literacy. A number of common themes were identified, as are presented in Table 6. While it could be argued the items listed under the themes presented in Table 6 above align with some of the existing physical literacy components articulated in Table 1, the fact that participants felt them important to mention suggests they represent culturally important language around components, worth considering as particularly relevant in the physical literacy context for the island of Ireland.

The vision of physical literacy as a concept supporting lifelong engagement not only in physical activity, but also in society, is notable, particularly when considering participants in this study were those working within the youth context. This highlights that although participants work within a youth sector, they recognise their role, and the role of physical literacy, as a lifelong influence. The lifelong nature of physical literacy is consistently a core defining feature of the concept, although the majority of research to date has been predominantly focussed on a youth context [54]. It is encouraging to see that stakeholders themselves working in this youth context see the importance of a proactive and salutogenic approach to promoting physical literacy and it would suggest that there is a readiness amongst key stakeholders to adopt an approach to physical literacy policy development that involves a ‘life-long’ physical literacy journey’

Findings from the current study, in line with the Australian consensus statement [5], and work by Mandigo et al. [43] in Canada , highlight the social domain as a key component of physical literacy. Various social benefits of physical literacy, including ‘social skills’, ‘relating to others’, ‘competent within society for life’, ‘participating in a meaningful way in society’ and ‘becoming a better citizen’, were all identified by participants in this study. This emphasis on social benefits of physical literacy differs from the dominant International Physical Literacy Association definition [44], who do not currently include the social as a separate named domain or element within their definition. The growing recognition of the social element across different international perspectives suggests the social component of physical literacy is an area that warrants further consideration and research. With regard to the purpose of the current study, this finding suggests that to stakeholders on the island of Ireland there is a social context associated with physical literacy that is valued and is important to consider in consensus statement development work.

In addition, the potential element of physical literacy which speaks to ‘mind and body connectivity awareness’ is also important. This theme shows similarities with what Whitehead placed as one of the three underpinning philosophies of physical literacy; monism [1, 52]. While it has been suggested that the underpinning philosophy of physical literacy has been what has made the concept ‘*abstract and inaccessible*’ (pg 372, [11]), it is encouraging to find that whilst the terminology may differ, stakeholders value the intertwined importance of the mind and body together, which is often referred to as a holistic approach. This notion of physical literacy related constructs being intertwined and interdependent is further reinforced by the findings that suggested the difficulty in separating constructs, such as ‘knowledge and understanding’ and ‘movement competence’. The theme of ‘movement vocabulary and safety’ is also noteworthy. To a very large extent this could well be considered to fall under a heading of ‘knowledge and understanding’, however it is also true to say many people may view ‘knowledge and understanding’ from a much narrower and more cognitive paradigm. This raises the question for the development of the all-island consensus statement; whether the knowledge and understanding paradigm needs to be more clearly articulated and defined, or whether in fact an additional category needs to be included to capture this.

### Key considerations moving forward

In developing a consensus statement for the island of Ireland, and more particularly when operationalising this statement, emphasis will need to be placed on addressing the ‘poorer’ status of both the affective and cognitive domains when compared with the physical domain. Specifically, some of the ‘personal benefits’ highlighted by stakeholders when asked ‘What does physical literacy mean to you?’ (Table 6), including ‘mental attitude and strength’, ‘emotional and cognitive benefits’ and ‘fulfilment’, may suggest that there is some appreciation for the cognitive and affective aspects of physical literacy. A factor that can influence stakeholders’ perceptions is stakeholder understanding of the terminology used relating to physical literacy domains or components. Martin’s et al., [17] suggested that simplifying knowledge and terminology is important to help make physical literacy more accessible and usable by those who are directly involved with implementation in the fields of education, sport, and public health.

More accessible language may assist in addressing the imbalances currently evident in the prioritising of domains, and help remove ambiguity as to what each domain means (and what components it may represent). As previously discussed however, this is a balance, as there is a need to be mindful of the work of Young et al. [15] who warned that simplified interpretations of the concept risk uncoupling from the core meaning of the concept. Future work in disseminating the holistic nature of the concept in an accessible manner with practical guidance on developing all domains of physical literacy is needed – which has very direct implications for the development and operationalisation of Ireland’s all-island consensus statement on physical literacy. Potentially given the existing grasp of concepts linked to affective and cognitive evidenced in the data above, development of a more holistic approach to physical literacy will be akin to ‘pushing against an open door’ with key stakeholders who show some indications of understanding or ‘readiness’ to move to holistic approaches when describing PL in their own words. A crucial consideration moving forward (which was beyond the scope of the current study), would be to explore how stakeholders acquired their perceptions, as this knowledge could aid researchers in better translating physical literacy theory.

Crucially, findings of this study suggest that currently those working in education settings are more comfortable with concepts relating to physical literacy, and as such future work to grow expertise across all sectors will be vital. Findings also suggest that, currently the physical/psychomotor domain is better understood and prioritised amongst Irish stakeholders, and so efforts to develop and encourage a more holistic approach with specific emphasis on elevating the status of both the affective and cognitive (knowledge and understanding) domains is warranted; developing physical literacy as a lifelong journey is an important perception of the concept amongst stakeholders. Study findings support that there is a level of readiness for such developments. Consideration and adoption of the ‘social benefits’ of physical literacy also has importance to Irish stakeholders and deserves attention in the development physical literacy across the island.

### Strengths and limitations

Strengths of the current study include the nationally representative sample, from NI and the RoI, and representing a broad range of stakeholders involved in delivering physical literacy related programmes to young people. As stated, it was beyond the scope of this project to focus on other life stages, although the importance of physical literacy as a lifelong journey was conveyed in responses. This is the first study that this research team is aware of to investigate and incorporate a range of stakeholders perceptions of physical literacy in the formative stages of national consensus statement development. The information obtained regarding how physical literacy is understood, how understanding differs, and indeed the priority placed on the different domains of physical literacy by stakeholder groups, provides critical information which can inform the development of Ireland’s all-island consensus statement. Early stakeholder involvement can hopefully prevent some of the ‘uncertainty, confusion, and resistance’ (pp.1 [55]) to the concept, and this involvement should be ongoing. Acknowledging that physical literacy is already being developed and operationalised, this work is building on already established practices that are embedded across the social, cultural environments.

The researcher developed questionnaire used in this study is a limitation, as no existing validated questionnaire was available to meet the needs of the study. Nevertheless, the data gathered in this study supports the development of a statement for physical literacy for the island of Ireland, that is culturally and socially relevant, and provides an insight into how physical literacy is interpreted, how important various components are perceived to be, and how it is prioritised by those working in various roles and sectors on the island of Ireland. The open-ended question item, which was posed at the start of the survey was optional, with 48% of participants choosing to respond. While a lower response rate to open ended questions due to burden placed on participants is to be expected [53], it is possible also that it could due to other factors in addition to participant burden, including a potential lack of confidence or ability of some participants in articulating understanding of the concept. Responses offered are nonetheless very useful, however should be interpreted with caution.

## Conclusions

This study is one of the first to engage with ‘on the ground’ stakeholders to gather their perceptions regarding physical literacy, with a view to informing the development of a physical literacy statement. This study included a wide array of potentially relevant physical literacy components, thus providing a broad and comprehensive investigation of participants understanding and perceived importance of same. Findings demonstrate that different stakeholder groups have a wide variation of expertise and experience in physical literacy, and place different levels of importance on the various physical literacy domains/components; underlining the need for the all-island statement on physical literacy to ‘understand the audience’ and to continue to engage with stakeholders, to ensure that the statement is effectively operationalised in a cross- sectoral, collaborative manner.

This paper offers valuable new insights, which have not been published related to previous physical literacy consensus statements internationally. The definitions, interpretations and level of importance placed upon physical literacy in society will continue to transcend, as the evidence for policy level physical literacy implementation improves. The stakeholder data from the RoI and NI that informed the current study has critically evaluated physical literacy as a holistic construct, and presents cutting-edge information on societal perspectives for those working with children and youth, and critically, informs the development and operationalisation of the all-island consensus statement on physical literacy. Continued engagement with stakeholders during the development of the statement, and throughout its operationalisation, is critical to achieve sustained, meaningful and impactful implementation. These findings lay an important foundation, but the operationalisation, and evaluation of the implementation of the resulting statement is key [17]. This ongoing process will ultimately contribute towards a goal that is shared amongst many stakeholders working within physical literacy; to empower individuals to choose physical activity for life.

## Data Availability

The datasets used and/or analysed during the current study are available from the corresponding author on reasonable request.

## Abbreviations

CSPPA: Children’s Sport Participation and Physical Activity Survey
NI: Northern Ireland
PE: Physical Education
RoI: Ireland
SI: Sport Ireland
SNI: Sport Northern Ireland
